# Endoluminal dilatation for embedded hemodialysis catheters: A case-control study of factors associated with embedding and clinical outcomes

**DOI:** 10.1371/journal.pone.0174061

**Published:** 2017-03-27

**Authors:** Hari Talreja, Stephen Edward Ryan, Janet Graham, Manish M. Sood, Adnan Hadziomerovic, Edward Clark, Swapnil Hiremath

**Affiliations:** 1 Division of Nephrology, Faculty of Medicine, University of Ottawa, Ottawa, Ontario, Canada; 2 Department of Medical Imaging, Faculty of Medicine, University of Ottawa, Ottawa, Ontario, Canada; 3 Clinical Epidemiology Program, Ottawa Hospital Research Institute, Ottawa, Ontario, Canada; Postgraduate Medical Institute, INDIA

## Abstract

**Background:**

With the increasing frequency of tunneled hemodialysis catheter use there is a parallel increase in the need for removal and/or exchange. A small but significant minority of catheters become embedded or ‘stuck’ and cannot be removed by traditional means. Management of embedded catheters involves cutting the catheter, burying the retained fragment with a subsequent increased risk of infections and thrombosis. Endoluminal dilatation may provide a potential safe and effective technique for removing embedded catheters, however, to date, there is a paucity of data.

**Objectives:**

1) To determine factors associated with catheters becoming embedded and 2) to determine outcomes associated with endoluminal dilatation

**Methods:**

All patients with endoluminal dilatation for embedded catheters at our institution since Jan. 2010 were included. Patients who had an embedded catheter were matched 1:3 with patients with uncomplicated catheter removal. Baseline patient and catheter characteristics were compared. Outcomes included procedural success and procedure-related infection. Logistic regression models were used to determine factors associated with embedded catheters.

**Results:**

We matched 15 cases of embedded tunneled catheters with 45 controls. Among patients with embedded catheters, there were no complications with endoluminal dilatation. Factors independently associated with embedded catheters included catheter dwell time (> 2 years) and history of central venous stenosis.

**Conclusion:**

Embedded catheters can be successfully managed by endoluminal dilatation with minimal complications and factors associated with embedding include dwell times > 2 years and/or with a history of central venous stenosis.

## Introduction

Access to the vasculature, afforded since the advent of the Quinton-Scribner shunt and the cannulation of the central venous system, has made hemodialysis possible for the last half century[1]. Given the problems with infectious and mechanical complications, as well as the higher mortality associated with them, central venous catheters (CVC) are reserved only for patients who are unable to start hemodialysis with a permanent access, i.e. an arteriovenous fistula or graft. The realities of patient co-morbidity, however, result in a substantial proportion of hemodialysis patients with a CVC at any given time as their vascular access[2], which makes the issue of preventing and treating complications with CVCs, as they occur, of paramount importance.

Recent publications have described cuffed, tunneled hemodialysis catheters that were difficult to remove after the standard cutdown and traction method, and are reported as being tethered, embedded or ‘stuck’[3, 4]. The exact pathophysiology of this phenomenon is not known, but is thought to be due to adhesion of the fibrous sheath, which usually forms over the catheter, to the vessel wall. In these cases, the fibrous sheath may adhere to the superior vena cava which makes removal by traditional methods difficult and dangerous[5, 6]. The approach to this complication has varied in the reported literature, from one of cutting the external part of the catheter and burying the rest in a subcutaneous pocket thus leaving the intravascular component *in situ*, to that of surgical removal after cardio-pulmonary bypass[7, 8]. Another group has described catheter removal using a laser sheath to excise the adhesions by a cardiothoracic surgeon with a technique modified from the one to remove pacemaker/defibrillator leads[9]. Our group has previously described successful removal of 5 catheters without any complications after endoluminal balloon dilatation[10]. Endoluminal dilatation was done in the interventional radiology suite under local anesthesia and conscious sedation, intact catheters were removed successfully and all patients were discharged the same day.

Leaving the catheter buried *in situ* exposes patients to infection, mechanical thromboembolism, chronic pain and anxiety. Since operative removal is an invasive procedure and would be a high risk procedure for most hemodialysis patients, one of the suggested approaches is to change CVCs regularly to preemptively prevent this complication[8]. Our preliminary experience of removing tethered catheters by endoluminal balloon dilatation appears safe and effective, however to date there are limited reported outcomes and complications related to this procedure. [10]. In this case series we describe the patient outcomes associated with endoluminal dilatation for embedded catheter removal and try to identify risk factors for this complication using a case-control study design.

## Methods

### Study design and data collection

We performed a case control study to identify patient factors which associate with tethered catheters. Ethics approval was obtained from the institutional review board (Ottawa Health Sciences Network Research Ethics Board) prior to study initiation. Since this was a retrospective study based on chart review, and had no patient contact, consent was waived. All patients who had embedded catheters from January 2010 were identified by our vascular access coordinator (JG). We selected controls (at a 1:3 ratio of cases to controls) as those patients who had undergone uncomplicated catheter removal during the same period. The electronic medical record, Nephrocare (Fresenius Medical care, Bad Homburg, Germany) was used to retrieve patient characteristics. We collected baseline data on patients (demographics, co-morbidities and medication use) and catheters (radiographic history of central venous stenosis, previous CVC insertions the reason for catheter removal and the duration the catheter was *in situ*). The reasons for catheter removal were grouped as related to complications (either infectious such as bacteremia or exit site or mechanical such as poor function) or related to change of modality or access (transplant, change to peritoneal dialysis or maturation of permanent access).

### Details of endoluminal dilatation

Endoluminal dilation procedures were performed in an interventional radiology suite by fellowship-trained interventional radiologists. All procedures were performed under sterile conditions and under both local anesthesia (1% lidocaine) and intravenous sedation (midazolam, fentanyl) with patients continuously monitored with electrocardiography and pulse oximetry.

**Fig 1:**
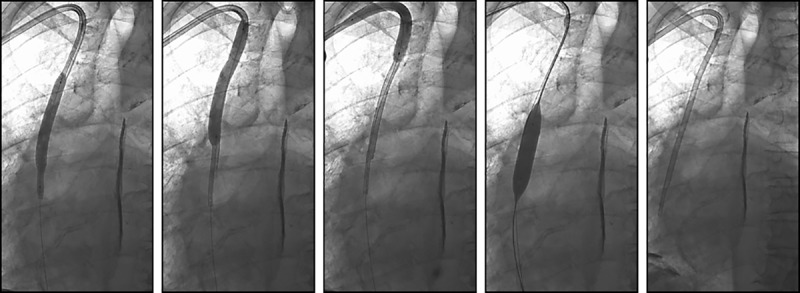
Endoluminal Dilation: (A, B and C) An 8 x 80mm balloon is inserted over a guidewire into the venous lumen of an embedded tunneled dialysis catheter and inflated along the length of the catheter from its distal tip to the cuff. Balloon inflation pressure of between 12 (rated burst pressure) and 20atm is used and maintained until there is complete or near complete effacement of areas of narrowing, assumed to represent the principle points of tethering of the catheter to the venous wall. The catheter is then easily removed. (D and E) If performing a catheter exchange, a 0.035inch guidewire is inserted through the arterial lumen of the embedded catheter just before its removal and used for insertion and inflation of a 12 x 40mm balloon along the length of the SVC and brachiocephalic vein. The balloon is then removed followed by insertion of a new tunneled dialysis over the guidewires and through the initial subcutaneous tract.

The cuff of the indwelling tunneled dialysis catheter was first dissected from its subcutaneous tunnel. When removal of the catheter failed despite forceful retraction, endoluminal dilation was performed. The first few dilation procedures were performed using 0.035 inch guidewire and balloon catheters. The technique was subsequently modified as follows (see Fig 1). A 0.018-inch x 150cm SV-5 wire (Cordis, Milpitas, California) is inserted through the venous lumen and navigated into the IVC. An 0.035-inch x 180cm regular or stiff shaft Glidewire (Terumo, Somerset, New Jersey) is then optionally placed into the superior vena cava (SVC) through the arterial lumen to be used as both a safety wire and to aid in catheter exchange if required. An 8 x 80mm Sterling balloon (Boston Scientific, Marlborough, Massachusetts) on an 80cm shaft is subsequently inserted into the venous lumen over the SV-5 guidewire and advanced to near the distal tip of the dialysis catheter. Using an Encore 26 inflation device (Boston Scientific), the Sterling balloon is inflated to its rated burst pressure (12atm) and maintained at this pressure until there is complete or near complete effacement of areas of narrowing. On occasion, inflation pressures as high as 20 atm were required to achieve uniform balloon dilation. Endoluminal dilation is repeated along the length of the catheter up to the cuff. If the catheter is not then easily removed, dilation of the entire venous lumen is repeated. Once the catheter has been removed, sterile dressings are applied. If a catheter exchange was requested due to poor catheter function, the SVC and brachiocephalic vein are first dilated using a 12mm diameter Mustang balloon (Boston Scientific) followed by insertion of a new tunneled dialysis catheter over the guidewire and through the initial subcutaneous tract.

### Data analysis

Summary descriptive statistics are presented for the collected data. Where indicated, data are presented as the mean ± standard deviation (for continuous variables) and proportion (for categorical variables). Univariate comparison between cases and controls was performed using the student’s t-test for continuous variables and the chi-square test for categorical variables. Logistic regression was performed using prespecified covariates (diabetes, age, smoking history, gender, history of vascular disease, prior CVC, history of central vein (CV) stenosis, duration and side of CVC and cause[complications versus modality change] of catheter removal) to identify significant risk factors for embedded catheter formation. Duration of catheter was categorized as the proportion of patients with duration > 2 years for adjusted analysis. Analysis was performed using JMP (version 8.2, SAS Inc, Cary, NC). A two-tailed p value of 0.05 was considered significant for all analysis.

## Results

We identified 15 patients who had tethered CVCs and had required endoluminal balloon dilatation for catheter removal (‘cases’) who were matched 1: 3 with 45 patients who had undergone uncomplicated CVC removal at the bedside using the standard method (‘controls’). The demographic characteristics of cases and controls are presented in Table 1.

**Table 1:** Demographic characteristics.

	Cases	Controls	p value
**N**	15	45	
**Age (in years; mean** **±** **sd)**	68.7 ± 15.1	64.4 ± 12.8	0.33
**Men**	9 (60%)	27 (60%)	0.99
**Body mass index (in kg/m**^**2**^**; mean** **±** **sd)**	26.5 ± 6.4	26.0 ± 5.4	0.82
**Never Smokers**	9 (60%)	22 (48.9%)	0.56
**Diabetes**	7 (46.8%)	23 (51.1%)	0.99
**Hypertension**	15 (100%)	39 (86.7%)	0.32
**Statin Use**	12 (80%)	29 (64.4%)	0.35
**Previous Tx**	3 (20%)	4 (8.9%)	0.35
**Coronary artery disease (CAD)**	8 (53.3%)	16 (35.6%)	
**Cerebrovascular disease (CVD)**	4 (26.7%)	3 (6.7%)	
**Peripheral vascular disease (PVD)**	3 (20%)	5 (11.1%)	
**Any Vascular Disease**	8 (53%)	19 (42%)	0.55

All values in N (%) except as specified

Baseline characteristics were similar between the two groups with non-statistically significant differences in age (embedded CVC mean age 69 years vs 64 years in controls), statin use (80% vs 64% in controls) and peripheral vascular disease (20% vs 11% in controls). The reasons for CVC removal is presented in Table 2. CVC complications (i.e. infections and mechanical problems) were more likely to be the reason for catheter removal in cases versus controls (87% versus 35%) though the difference was not significant (p = 0.19).

**Table 2:** Causes for catheter removal.

*Cause of removal*	Cases	Controls	combined causes	p value
**Infection**	6 (40%)	10 (22.2%)	86.7% vs 35%	0.19
**Poor Blood Flow**	4 (26.7%)	6 (13.3%)		
**Extrusion of Cuff/Mechanical Issues**	2 (13.3%)	0		
**Stopping Dialysis**	1 (6.7%)	0		
**Recovery**	0	5 (11.1%)	13.3% vs 65%	
**Other Functional Access**	2 (13.3%)	15 (33.3%)		
**Switch to peritoneal dialysis**	0	9 (20%)		

All values in N (%)

The vascular access history is presented in Table 3. There was a trend towards higher rates of prior CV stenosis in cases (27% versus 13%, p = 0.06), left sided CVCs (26.7% versus 8.9%, p = 0.1) and higher rates of prior indwelling CVCs (67% versus 44%, p = 0.23). The duration of CVC was significantly greater amongst cases than controls when treated as a continuous variable (average duration 1505 days versus 256 days, p <0.001) or a nominal variable (duration > 2 years, n = 14, 93% in cases versus n = 2, 4% in controls, p < 0.001).

**Table 3:** Access characteristics.

	Cases	Controls	p value
**Left side**	4 (26.7%)	4 (8.9%)	0.10
**h/o Past CVC**	10 (66.7%)	20 (44.4%)	0.23
**h/o CV stenosis**	4 (26.7%)	3 (6.7%)	0.06
**Duration of line (in days, mean** **±** **sd)**	1505 ± 632	256 ± 223	<0.001
**thrombolytic agent use**	8.3 ± 19.2	9.2 ± 25.8	0.91
**duration > 1 year**	100%	20%	<0.001
**duration > 2 years**	93.3%	4.4%	<0.001
**duration > 5 years**	73.3%	0%	<0.001

All values in N (%) except as specified

In the multivariable analysis, only past CV stenosis (p < 0.03) and duration > 2 years (p < 0.001) were significant predictors for embedded catheters.

The catheter removal with endoluminal dilatation was uncomplicated in all the 15 cases, with no post-removal sepsis, surgical complications or mortality. The entire catheter was removed with in all cases with no fragments left *in situ*.

## Discussion

In the largest case series to date involving endoluminal dilatation for embedded catheter removal, we found the procedure was well tolerated and with no discernable complications. All cases attempted were successful. Factors associated with embedded catheters included longer duration of CVC *in situ* and a previous history of central venous stenosis. These findings illustrate that endoluminal dilatation provides a safe potential alternative to traditional management strategies.

In addition to our present and previous report, we found 12 citations which reported tethered, embedded or stuck tunneled catheters in a total of 50 hemodialysis patients (Table 4)[3, 4, 7–17]. The gender distribution was comparable (45% men) and the age range varied from 20 to 82 years. 9 (25%) of the 36 internal jugular catheters were left sided. The duration of the catheters (from insertion to attempted removal) ranged from 11 months to 10 years. Sepsis (4/9, 44%) and a high rate of mortality (3/9, 33%) were reported in the catheters which were cut and buried.

**Table 4:** Review of literature.

Study	N	Gender (F: female; M: male)	Age (years)	Catheter site*	Duration	Technique	Complications
Thein 2005	1	F	53	L IJV	2.5 years	Cut and buried	Sepsis, Died
Hassan 2006	6	5F; 1M	Range 31–63	varied	3–7 years	All 6 cut and buried; 2 later removed surgically	1 died sepsis; 1 sepsis, treated.
Foley 2007	1	F	67	R IJV	7 years	Endovascular removal with snares; small part kept in situ	No procedural complications
Liu 2007	8	NR	NR	NR	1 to 10 years	2 removed surgically, rest cut and buried	NR
Field 2008	6	2F; 4M	24–82	3 R IJV; 3 L IJV	11–72 months	5 removed surgically; 1 cut and buried	Cut and buried: died of sepsis
Akgun 2008	2	2F	44 and 52	R IJV	27/55 months	Open surgical removal	no post-op complications
Carillo 2009	3	All men	NR	R IJV, L IJV, L SC	NR	Laser sheath	no procedural complications
Hong 2010	1	F	62	R IJV	2.5 years	Sheath	No procedural complications
Lopes 2010	1	F	79	R IJV	10 years	Cut and buried	none, maintained on prophylactic antibiotic
Hong 2011	1	F	74	R IJV	2.5 yrs	Balloon in OR	no procedural complications
Ryan 2011	6	*Included in present study*					
Farooq 2012	1	F	54	R femoral	11 months	Balloon dilatation	
Beigi 2013	4	2F; 2M	20–52	R IJV	NR	1 surgically removed; 1 removed endovascularly with IVC access, snare	2 died before removal; 1 developed sternomyelitis post-op, survived
Present Study	15	6F; 9M		4 L IJV 11 R IJV		Endoluminal balloon dilatation	none
**Summary**	**50**	**23F; 19M; 8 NR**	**Range 20 to 82 years**	**9 L IJV 25 R IJV 1 L SC 1 R Femoral, 14 NR**	**Range 11 months to 10 years**		

* R: right; L: left; IJV: internal jugular vein; SC: subclavian; NR: not reported

In comparison to previous cases reported in the literature, there were no surgical or infectious complications, or mortality, observed in these patients. From the reported literature, the approach of cutting and burying the catheter seems to be strongly associated with infections, sepsis and mortality, as would be expected. Moreover, the other method of removing the catheter surgically may be technically successful and may not be accompanied by infectious complications, but the nature of the surgical procedure is quite invasive and resource-intensive compared to our technique.

In the absence of widely recognized techniques for removal of such embedded catheters, a strategy of pre-emptive catheter exchange has been recommended[8]. Though such a strategy will likely prevent catheters becoming embedded and stuck, it would be quite resource-intensive and would be a substantial burden on the hemodialysis patients. Given that our technique has been safe and effective with no complications in a series of 15 patients, we suggest that it is no longer necessary to recommend this strategy of pre-emptive catheter exchange. Data from our case control study suggests that the combination of a long duration of catheter implantation (> 2 years) or history of central vein stenosis is associated with embedded catheters and could be used to triage patients to monitor for catheter removal, when clinically indicated, in the interventional radiology suite.

This complication also highlights the need to reduce catheter dependence in hemodialysis patients, as has been advocated by all professional societies and more recently spearheaded by the Fistula First Initiative[18, 19]. Unfortunately, for various reasons including patient factors[2, 20], long term catheter use remains quite common, and, as a consequence, the need to recognize and appropriately handle embedded catheters remains of paramount importance.

It is still not clear as to the exact mechanism of this phenomenon since all patients with a prolonged duration of catheter implantation do not develop this complication. Previous studies of non-embedded central venous catheters, from animal as well as human autopsy studies, suggest that this may be due to adhesion of the fibrous sheath that forms over the catheter to the vessel wall[5, 6]. Theoretically, if the catheter material is more biocompatible or results in a decreased tissue fibrous reaction, this could decrease the risk of this complication. We failed to find any other patient specific factors (such as gender, smoking status, use of statins or thrombolytics) that could describe this risk. Though left sided catheters were previously reported as a suspected factor, and also seemed to be somewhat higher (27% versus 9% in controls), after adjustment for duration of catheter and underlying central vein stenosis, this was no longer significant.

Our analysis, especially the adjusted analysis for risk factors of embedded catheters, is limited by the small number of cases, and should be considered hypothesis-generating at best. However, it does represent the largest case-series so far and probably represents the best available evidence on this topic.

In conclusion, we describe 15 cases of uncomplicated and successful removal of embedded tunneled catheters in hemodialysis patients. Prolonged duration of catheter implantation (> 2 years) and history of previous central vein stenosis are significant risk factors identified from our case control study.
